# The therapeutic effects of yoga in people with Parkinson’s disease: a mini-review

**DOI:** 10.1080/07853890.2023.2294935

**Published:** 2023-12-18

**Authors:** Ting Zhang, Wei Liu, Qingping Bai, Song Gao

**Affiliations:** a College of Physical Education and Health Sciences, Zhejiang Normal University, Jinhua, China; b University Hospital, Zhejiang Normal University, Jinhua, China; c Physical Education College, Guangxi University of Science and Technology, Liuzhou, China

**Keywords:** Parkinson’s disease, yoga, movement disorders, balance disorders, emotional disturbance

## Abstract

Parkinson’s disease (PD) is one of the most common neurodegenerative diseases, second only to Alzheimer’s disease. Drugs and deep brain stimulation (DBS) are the main treatments for PD. However, the long-term side effects of drugs and the risks of surgery cannot be ignored. It is therefore important to research safe and effective complementary and alternative therapies for PD. Yoga, an ancient mind-body exercise, has been widely used in health promotion. Although, yoga can address a range of health problems, little is known about its role in people with PD. This article reviews the evidence that yoga improves PD symptoms, including movement disorders, balance function and emotional disturbance. The authors analyze the role and shortcomings of the yoga intervention process, with the aim of providing a scientific basis for the application of yoga training to people with PD.

## 1. Introduction

Parkinson’s disease (PD) is one of the most common neurodegenerative diseases, second only to Alzheimer’s disease [1]. Resting tremor, bradykinesia, myotonia, and postural balance disorders are typical motor symptoms of PD [2]. In addition, non-motor symptoms, such as anxiety, depression, and sleep disorders, often occur unpredictably during the course of the disease [3]. The incidence of PD is positively correlated with age. As the world’s population ages, the number of people with PD is increasing rapidly. The number of PD patients worldwide exceeded 6 million in 2016 [4]. The number of PD patients in China is expected to reach 5 million by 2030, accounting for more than half of the world’s cases [5]. PD places a heavy burden on society and families. However, there is no effective treatment for PD. Drugs and DBS surgery can only provide limited symptom relief. The side effects of long-term medication cannot be ignored, such as postural hypotension, nausea, involuntary movements, falls, and sleep problems [6]. Also, patients with PD are skeptical about the technical risks and high cost of DBS. Thus, it is therefore important to explore safe and effective complementary alternative treatments for PD.

Yoga, as a mind-body therapy, is an important part of complementary and alternative therapies [7]. Yoga, derived from the Sanskrit word “yuj” or “yug”, is a sport that originated in India. Yoga emphasizes the integration of easily mastered physical skills into postures, implying the philosophical ideas of “oneness” and “harmony”. Practitioners expect to achieve harmony between brain activity and body functions through yoga practice [8]. Currently, yoga is widely used in the adjunctive rehabilitation of various diseases, including chronic low back pain, rheumatoid arthritis, hypertension, coronary heart disease, breast cancer [9], and neurological disorders such as stroke, PD, multiple sclerosis, spinal cord disease, traumatic brain injury, dementia and epilepsy [7]. Yoga has developed over a long period of time into different schools such as Janna Yoga, Karma Yoga, Bhaki Yoga, Raja Yoga and Hatha Yoga [10]. Hatha yoga is the most popular school of yoga in modern times, emphasizing a combination of asana practice, breathing techniques, and meditation, whereas other yoga types tend to favour either meditation or one of the breathing techniques [11]. Hatha yoga has evolved into therapeutic yoga systems such as Ashtanga yoga, Iyengar yoga, flow yoga, yin yoga, and hot yoga [12]. Yoga has been shown to have a positive effect on improving motor symptoms, balance and anxiety in people with PD [13].

Currently, yoga training for people with PD includes Hatha yoga [14], Iyengar yoga [15], flow yoga [16], or Ashtanga yoga [17], of which Hatha yoga is the most widely used, followed by Iyengar yoga. Settings for yoga training include rehabilitation centers, yoga studios, home settings, and community service centers [18]. The duration and frequency of yoga training for people with PD varied between studies. The duration and frequency of the yoga intervention is 45 or 60 min daily, twice a week [19]. The training cycle was usually 8 to 12 weeks. Although the duration, frequency, and training cycle of the intervention varied between trials, the effect of the intervention was recognizable. This review summarizes the current status of the use of yoga in people with PD, aiming to provide a reference base for improving the symptoms of PD. Characteristics of the included studies are shown in Table 1.

**Table 1.** Characteristics of the included studies.

References	Participants	Interventions	Outcomes
Cherup et al. [34]	*n* = 33 Age: 40–90 y HY scale 1–3 stage ability to walk with/without aids	A novel yoga meditation program vs. proprioceptive training 45 min, 2 times/wk, 12 wks	TUG
Cheung et al. [26]	*n* = 20 Age: 49–75 y HY scale 1–3 stage ability to walk with/without aids	Hatha Yoga vs. usual care 60 min, 2 times/wk, 12 wks	UPDRS, MoCA, Beck Depression Inventory
Colgrove et al. [22]	*n* = 13 Age: 50–88 y HY scale 1–2 stage ability to walk with/without aids	Yoga exercise vs. yoga sessions 60 min, 2 times/wk, 12 wks	UPDRS-III, BBS, ROM, muscle strength gait (postural sway and gait initiation
Jojo et al. [38]	*n* = 138 Age: 63.6 ± 8.7 y HY scale 1–3 stage ability to walk with/without aids	Mindfulness Yoga vs. stretching and resistance 60 min /wk, 8 wks	HADS, UPDRS-III, TUG, HWS, PDQ-8
Khuzema et al. [29]	*n* = 27 Age: 68.11 ± 4.23 y HY scale 2.5–3 stage ability to walk with/without aids	Modified Yoga vs. conventional balance exercise 30–40 min/wk, 8 wks	BBS, TUG, 10-Minute Walk test
Myers et al. [31]	*n* = 26 Age: 65.0 ± 8.7 y HY scale 2–3 stage ability to walk with/without aids	Modified Yoga vs. usual daily routines 60 min, 2 times/wk, 12 wks	BESTest, BAI
Ni et al. [23]	*n* = 23 Age: 72.2 ± 6.5 y HY scale 1–3 stage ability to walk with/without aids	High-speed yoga vs. 1-h nonexercise, health education 60 min, 2 times/wk, 12 wks; 1/mo × 3mo	UPDRS-III, BBS, Mini-BESTest, TUG, PDQ-39
Walter et al. [45]	*n* = 27 Age: 67.74 ± 5.89 y HY scale 1.5–3 stage ability to walk with/without aids	Modified Yoga vs. usual care 60 min, 2 times/wk, 8 wks	UPDRS, Mini-BESTest FGA, FOG, FS-16, PDQ-8

HY: Hoehn and Yahr scale; min: minute; wk: week; mo: month; TUG: timed up and go test; UPDRS: unified Parkinson’s disease rating scale; UPDRS-III: the motor subscale of the unified parkinson’s disease rating scale; BBS: Berg balance scale; PDQ-39: Parkinson’s disease questionnaire-39; BDI: Beck depression inventory; HADS: hospital anxiety and depression scale; MoCA: montreal cognitive assessment; ROM: range-of-motion; HWS: holistic well-being scale; PDQ-8: Parkinson’s disease questionnaire-8; Mini-BESTest: mini-balance evaluation systems test; BAI: beck anxiety inventory; FGA: functional gait assessment; FoG: freezing of gait questionnaire; PFS-16: parkinson’s fatigue scale-16.

## The effect of yoga in PD rehabilitation

### The effect of yoga on dyskinesia in people with PD

Typical motor symptoms in people with PD include tremor, muscle hypertonia, and bradykinesia. Movement disorders limit the functional independence of people with PD and seriously affect their quality of life [20]. Studies have shown that yoga can improve dyskinesia and daily activities in people with PD [21].

Colgrove et al. [22] randomized 20 people with PD into a yoga group and a control group. The yoga group received Iyengar yoga, which included relaxation exercises, yoga postures and meditation. The intervention was done twice a week for 60 min for 12 weeks. The control group continued to receive conventional care. The results showed that those in the yoga group had significant improvements in lower limb muscle strength, range of motion and flexibility.

Another study compared the effects of long-term power yoga (Ashtanga yoga) and conventional therapy on motor performance in PD patients [23]. It was found that power yoga not only reduced patients’ symptoms such as bradykinesia and stiffness in the upper and lower limbs, but also increased muscle strength in the lower limbs. There were no adverse events after the intervention. Stretching exercises and specific postures in yoga practice can lengthen muscle groups and activate stretch receptors in muscles, ligaments, and joints, which is beneficial for improving joint flexibility in the lower back, knees, and ankles [24,25]. This may be why yoga improves motor symptoms and increases stability in people with PD.

Cheung et al. [26] conducted a study in which 20 patients with PD were randomised to a yoga training group or a wait-list control group. The yoga group participated in 60-minute group sessions twice a week for 12 weeks. There was no significant difference in blood markers of oxidative stress between the two groups. Although the yoga group had better motor function scores on the Parkinson’s Disease Unified Rating Scale, they had worse sleep and outlook scores on the Parkinson’s Disease Quality of Life (PDQUALIF) scale and physical activity scores on the Longitudinal Aging Study Amsterdam Physical Activity Questionnaire compared to the control group. At the end of the yoga intervention program, motor function, cognitive function, and catalase improved. However, physical activity levels and the three PDQUALIF domains, which include social and role functioning, sleep, and outlook, deteriorated compared to baseline. As a complementary approach, yoga may be feasible and acceptable for improving motor function in patients with PD. Studies with larger sample sizes are needed to determine its effects on oxidative stress and non-motor symptoms.

The above studies suggest that yoga training has the potential to improve overall motor function and coordination in people with PD, for instance, by increasing muscle strength and joint flexibility. Nevertheless, the current studies have mostly been carried out on small samples, so more studies with larger samples are needed in the future, to strengthen the applications. It is important to get more clinical data to support the findings.

### The effect of yoga on balance disorders in people with PD

Maintaining balance is fundamental for normal gait and activities. As PD progresses, balance problems become progressively worse. The incidence of falls due to balance problems in PD patients ranges from 38% to 73%, increasing the risk of fractures [27]. Yoga exercises emphasize the conscious control of the body’s position in space, which effectively enhances the patient’s proprioceptive sensitivity, the stability of the vestibular function, and the comprehensive analysis ability of the cerebral cortex. Yoga training can enhance the level of postural control and improve balance in PD patients [28]. Khuzema et al. [29] showed that PD patients can use Tai Chi, yoga, or traditional balance exercises as therapeutic interventions to optimize balance and mobility.

Elangovan et al. [30] investigated the effects of Hatha yoga on gait and balance in patients diagnosed with PD. The patients’ functional mobility, postural stability, functional range of motion, and gait kinematics were assessed both before and after the intervention. People with PD experienced a significant decrease in the centre of mass sway path during standing after yoga training, suggesting an improvement in static balance. However, the study did not show a significant improvement in gait after yoga training, which could be due to insufficient intensity and dosage of the intervention.

Myers and colleagues [31] randomized patients with PD into two groups: a yoga group and a control group, each consisting of 13 participants. During the 12-week intervention, the yoga group received instruction twice a week, while the control group continued with their regular activities. All participants were assessed before and after the intervention using the Balanced Evaluation System Test (BESTest), the Beck Anxiety Inventory (BAI), and the Revised Oswestry Disability Index (ROSW). Both the yoga group (Z= − 3.2, *p*=.001) and the control group (Z= − 2.1, *p* = 0.04) showed improvement in their total BESTest score. The control group did not report any significant change in the individual balance system, whereas the yoga group showed improvement in the following systems: stability limit/verticality (Z= − 2.3, *p* = 0.02), transition/anticipation (Z= − 2.5, *p* = 0.01), reactivity (Z= − 2.7, *p* = 0.008), and sensory orientation (Z= − 2.3, *p* = 0.02). The reduction in ROSW was observed exclusively in the yoga group (Z= − 2.1, *p* = 0.03). No significant changes in BAI scores were observed in either group. Based on the results, it can be hypothesized that yoga may improve balance and alleviate low back pain in people with PD. However, there is insufficient evidence to support its effectiveness in improving mood.

The innovative Yoga Meditation Program (YoMed) has been shown to significantly enhance dynamic balance in elderly individuals with a history of falls [32]. This program combines motor imagery and action observation with an established proprioceptive training (PRO) intervention [33]. There is currently no research that has investigated the effectiveness of the YoMed program in people with PD.

Cherup et al. [34] conducted a study comparing the effectiveness of a YoMed with a PRO program in measuring proprioception and balance of patients with PD. Thirty-three patients with mild to moderate PD were randomized to either the YoMed or the PRO program. The duration of both interventions was 12 weeks, with two 45-minute sessions per week. The results showed that the YoMed group had significantly better Joint Kinesthesia flexion and Tinetti Balance Scale scores than the PRO group, and the difference was statistically significant (*p* < 0.01). These results suggest that the integration of yoga and meditation may be a beneficial approach to improving balance and proprioceptive ability in people with PD.

### Effect of yoga intervention on mood disorders in PD patients

Non-motor symptoms such as constipation, anxiety, and depression may occur before a definitive diagnosis of PD is made. However, people with PD usually do not associate anxiety and depression symptoms with PD in the early stages of the disease [35]. Anxiety and depression have a direct impact on the mental health of people with PD and worsen their movement disorders. This vicious cycle has a negative impact on mental health and quality of life [36]. Yoga training involves practices, including breathing regulation and meditation, that can improve the physical and mental health of PD patients and reduce stress [37]. In recent years, there has been increasing research into yoga as a potential therapy for mood disorders in PD patients.

A randomized controlled trial was conducted by Jojo et al. [38] in 138 clinically diagnosed patients with idiopathic PD (aged ≥18 years) who were randomized 1:1 to either yoga group or a stretching and resistance training exercise (SRTE) group. The study aimed to investigate the effects of mindfulness yoga (MY), stretching and resistance training on anxiety and depression in patients with PD through a randomized clinical trial. The intervention group received yoga in 90-minute sessions and the control group received SRTE in 60-minute sessions over a period of 8 weeks. Primary outcomes included anxiety and depressive symptoms, which were assessed using the Hospital Anxiety and Depression Scale. Secondary outcomes included the Movement Disorder Society Unified Parkinson’s Disease Rating Scale [MDS-UPDRS, Part III] motor score, perceived mental distress and calmness, and health-related quality of life (HRQOL). Assessments were made at baseline, 8 weeks and 20 weeks. The results of the study showed that the Yoga program was as effective as SRTE in improving motor dysfunction and mobility, with the added benefit of reducing anxiety and depressive symptoms, and improving mental health and quality of life. Furthermore, the effects of intervention were still evident at the three month follow-up [38]. The majority of participants reported feeling calm and relaxed during the regulated breathing phase.

One study found that meditation improved sleep disturbances and maintained a stable mood in almost 40% of people [39]. The exact mechanism by which yoga improves mood disorders in PD patients is unclear. One possible explanation is that yoga training stimulates the vagus or parasympathetic nerves and increases endogenous dopamine release from thalamic gamma-aminobutyric acid [40,41]. Thus, mindfulness yoga training may be effective in improving negative emotions in patients with PD. Nevertheless, yoga for improving psychology and mood in PD patients has received less research attention, with most studies focusing mainly on improving motor dysfunction. Additional, high-quality clinical trials should be conducted in the future to investigate the potential mechanisms of yoga in improving anxiety and depression in patients with PD.

### The effect of yoga intervention on other symptoms in patients with PD

Non-motor symptoms, such as fatigue, sleep disturbance, and pain, have a significant impact on people with PD, as do motor symptoms. Yoga has the potential to improve non-motor symptoms, such as fatigue and sleep disturbance in people with PD. Mindfulness yoga can equip patients with PD with enduring skills for emotional and psychological cognitive adjustment and aid in managing negative emotions arising from symptom experiences [42]. A study conducted by Jojo and colleagues [43] examined the mediating role of negative emotions in the relationship between changes in non-motor and motor experiences and quality of daily life. The research findings suggested that mindfulness yoga has a noteworthy effect on non-motor experiences, with a moderate to significant impact size (Cohen’s d: T1 = 0.50, T2 = 0.68). Therefore, this research confirmed a need for increased psychological nursing interventions to alleviate the detrimental impact of PD on both motor and non-motor aspects. In line with the findings of both systematic reviews [2,44], mind-body exercise shows a moderate clinical effect on the health of individuals with PD.

In a study by Walter et al. [45], 27 PD patients were randomized to a yoga group or a wait-list control group. Participants in the yoga group (*n* = 15) showed improvements in motor function, postural stability, functional gait, and freezing gait. They also had a reduced risk of falling. Postural stability improved significantly in the wait-list control group (*n* = 12), although their risk of falling was not reduced. Yoga training for people with PD reduced symptoms of fatigue and limitations in activities of daily living, while increasing their confidence to actively balance and controll falls. In another randomized controlled trial [22], the yoga group had significantly improved UPDRS scores (*p* = 0.006), diastolic blood pressure (*p* = 0.036), and mean exertional lung capacity (*p* = 0.03). There were also between groups changes in both subscales of the SF-36. There were trends towards improvement in depression scores (*p* = 0.056), body weight (*p* = 0.056), and forced expiratory volume in one second (*p* = 0.059). Participants who practised yoga reported more positive changes in symptoms, including a reduction in immediate tremor. However, there are few studies on the effects of yoga on non-motor symptoms in people with PD. Therefore, more studies are necessary to explore this further in the future.

### Risk of bias within studies

**Figure 1.**
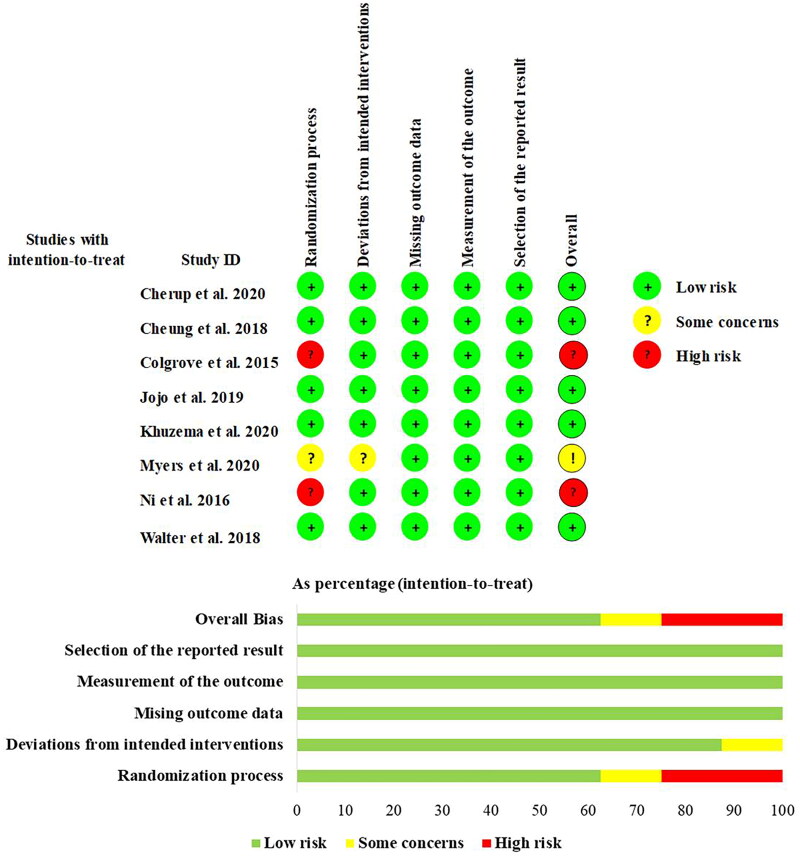
Risk of bias assessment.

The researchers used the Cochrane Risk of Bias Tool (version 2) to assess the methodological quality of the eight included trials. The overall quality of the study design was good, with 5 (62.5%) studies assessed as low risk of bias and 2 (25%) as high risk of bias, mainly due to bias in the randomisation process. For domain 1, 5 studies (62.5%) had a well-described randomisation process and were therefore assessed as low risk of bias, 1 study (12.5%) had some problems and 2 studies (25%) were at high risk of bias in this domain. Domain 2 assessed bias due to deviation from the intended intervention. 7 studies (87.5%) were assessed as having a low risk of bias and 1 study (12.5%) was assessed as having some risk of bias. Domain 3 assessed bias due to missing outcome data, and eight studies (100%) were assessed as low risk of bias. This means that each study obtained outcome data for almost all subjects. Domain 4 assessed bias due to the measurement of outcomes, and all studies were assessed as being at risk of bias. Domain 5 assessed bias in the selection of reported outcomes, and eight studies (100%) were assessed as being at low risk of bias. The methodological risk of bias in the included literature is shown in Figure 1.

### Precautions and safety in yoga practice

Yoga training often does not include all the asanas, and the movements may be simplified according to the characteristics of the disease [46]. Researchers should choose the appropriate type of yoga and asana in combination with the age of PD patients, stage of disease progression, degree of limb movement, etc. Patients with PD should choose yoga asana and exercise intensity appropriately during yoga training to avoid adverse events. The purpose, nature and precautions of the intervention should be fully explained to the patients before the yoga intervention. During the yoga training, the investigator or family members should closely monitor the patients’ physical status. If the patient becomes unwell, it should be stopped immediately and appropriate medical treatment should be given. No adverse events of yoga training were reported during the study period, or those that occurred during the study period were not related to the yoga intervention [31,34,38]. One study reported that patients with PD in both the yoga and control groups had mild knee pain [38]. In conclusion, early progressive yoga interventions in patients with PD are safe, but should be done under the supervision of a healthcare professional or specialised yoga instructor.

## Discussion and conclusion

Mind-body exercise includes a range of exercises such as mindfulness, meditation, yoga and tai chi that are commonly used in primary clinical practice [47]. A recent survey in the United States found that 83.1% of 5,000 people with PD considered exercise to be the most commonly used method to improve symptoms [48]. Of those who did exercise, 38.7% improved motor and non-motor symptoms with mindfulness exercise. A systematic review showed that tai chi reduced falls 6 months after the end of treatment [49]. Mind-body exercise can improve mental resilience by reducing psychological distress, which in turn improves quality of life in people with PD [50]. Mind-body exercise has received increasing attention in health promotion, and the evidence for its use in improving PD is beginning to accumulate.

Yoga, as a mind-body therapy, is beginning to be used in PD rehabilitation as a simple, adaptable and effective complementary alternative therapy [51]. Yoga training has been shown to be effective in improving motor symptoms, balance function and mood disturbances. Yoga interventions are most commonly delivered by professional yoga therapists, yoga-trained rehabilitation physicians and physiotherapists who work together to develop a programme and guide the patient through the exercises. Professionals from more than one of the above disciplines are involved in counselling through face-to-face groups or remote online interventions [38,45,52]. The target group is mainly Hoehn-Yahr stage I-III patients who are in the “open state” during the medication period and have some degree of balance and cognitive ability. Patients with PD Stage IV or V with more severe motor disabilities, it is difficult to complete the specific postures in yoga training, in the absence of ensuring the safety of the situation is not recommended to perform yoga. Further research is needed to understand the physical and psychological benefits of yoga as a multi-component physical activity or as an individual therapy for different stages of PD. During yoga training, participants should complete the appropriate amount of training according to their level of physical activity to avoid adverse events. More specific yoga poses should be adapted by the rehabilitation therapist and instructor to the patient’s actual condition.

Although all studies have shown beneficial effects of yoga on the control of motor and non-motor functions, there are methodological limitations to these studies. First, most of the existing studies were single-centre, small sample studies that could not rule out bias in the study design, which may affect the accuracy of the results. Multicentre, large, high-quality randomised controlled trials are needed to further investigate the effects of interventions. Secondly, patients with PD received different types of yoga training, and fewer comparisons were made between the effects of different yoga training. Thus, there is a need for researchers to conduct studies comparing the effects of different schools of yoga, to explore more appropriate movements for people with PD. Finally, there is a lack of normative standards for the implementation of yoga training. A unified practice guideline should be developed to guide relevant healthcare professionals in their clinical practice to promote rehabilitation therapy for patients with PD.

Yoga as a clinical intervention has not been widely promoted and used in the treatment of PD [53]. As people with PD are often treated at home or in the community, remote yoga training *via* the internet platform will help more people with PD to manage their condition. Moreover, few studies have monitored and followed up the long-term effects of the intervention. Post-intervention follow-up data should continue to be collected after yoga training to assess the effectiveness, stability, and duration of the intervention.

## Data Availability

Data sharing is not applicable to this article, given that no new data were created or analysed in this study.
